# Metformin is a novel suppressor for transforming growth factor (TGF)-β1

**DOI:** 10.1038/srep28597

**Published:** 2016-06-28

**Authors:** Han Xiao, Jianshu Zhang, Zhonghe Xu, Yenan Feng, Mingliang Zhang, Jianli Liu, Ruifei Chen, Jing Shen, Jimin Wu, Zhizhen Lu, Xiaohong Fang, Jingyuan Li, Youyi Zhang

**Affiliations:** 1 Institute of Vascular Medicine, Peking University Third Hospital and Academy for Advanced Interdisciplinary Studies, Peking University, Key Laboratory of Cardiovascular Molecular Biology and Regulatory Peptides, Ministry of Health, Key Laboratory of Molecular Cardiovascular Sciences, Ministry of Education and Beijing Key Laboratory of Cardiovascular Receptors Research, Beijing 100191, China; 2 Key Laboratory for Biomedical Effects of Nanomaterials and Nanosafety, Institute of High Energy Physics, Chinese Academy of Sciences, Beijing 100049, China; 3 Beijing National Laboratory for Molecular Sciences, Key Laboratory of Molecular Nanostructures and Nanotechnology, Institute of Chemistry, Chinese Academy of Sciences, Beijing, 100190, China

## Abstract

Metformin is a widely used first-line antidiabetic drug that has been shown to protect against a variety of specific diseases in addition to diabetes, including cardiovascular disorders, polycystic ovary syndrome, and cancer. However, the precise mechanisms underlying the diverse therapeutic effects of metformin remain elusive. Here, we report that transforming growth factor-β1 (TGF-β1), which is involved in the pathogenesis of numerous diseases, is a novel target of metformin. Using a surface plasmon resonance-based assay, we identified the direct binding of metformin to TGF-β1 and found that metformin inhibits [^125^I]-TGF-β1 binding to its receptor. Furthermore, based on molecular docking and molecular dynamics simulations, metformin was predicted to interact with TGF-β1 at its receptor-binding domain. Single-molecule force spectroscopy revealed that metformin reduces the binding probability but not the binding force of TGF-β1 to its type II receptor. Consequently, metformin suppresses type II TGF-β1 receptor dimerization upon exposure to TGF-β1, which is essential for downstream signal transduction. Thus, our results indicate that metformin is a novel TGF-β suppressor with therapeutic potential for numerous diseases in which TGF-β1 hyperfunction is indicated.

Metformin was originally derived from the French lilac *Galega officinalis*, and it is currently a widely prescribed biguanide used as a first-line antidiabetic drug. Metformin is safe and effective in the treatment of diabetes and does not induce hypoglycemia. Beyond its known blood glucose lowering effects, metformin has been shown to elicit beneficial effects on cardiovascular diseases^1^, polycystic ovary syndrome^2^, diabetic nephropathy^3^, and cancer^4^. However, the mechanisms underlying the pleiotropic effects of metformin remain elusive. Transforming growth factor-β1 (TGF-β1) is involved in the progression of many diseases, including cancer and fibrotic, cardiovascular, and immunological diseases. It is associated with fibrosis, epithelial-to-mesenchymal transition (EMT) and inflammation which are important pathological changes in the aforementioned diseases. Our previous study revealed that metformin inhibits cardiac fibrosis by inhibiting the TGF-β1-Smad3 signaling pathway^5^, which raises an intriguing possibility that metformin may prevent organ fibrosis via targeting TGF-β1 signaling. To date, the molecular and pharmacological mechanisms underlying metformin’s ability to inhibit the TGF-β1 signaling pathway are unknown, and identifying these mechanisms represents an important challenge that may elucidate new molecular targets of metformin and promote target-based novel strategies against many diseases beyond diabetes in which TGF-β1 signaling malfunctions are indicated.

The TGF-β1 signaling pathway requires two cell-surface serine/threonine kinase receptors, type II (TβRII) and type I (TβRI) TGF-β receptors. In general, TGF-β1 binds to TβRII, which recruits TβRI to form a heteromeric complex of TβRI-TβRII. The formation of this complex leads to the phosphorylation of TβRI and subsequent phosphorylation of receptor-regulated Smads (R-Smads, i.e., Smad2, Smad3, etc), which bind a co-Smad (Smad4). The R-Smad/co-Smad complexes translocate and accumulate in the nucleus and subsequently regulate target genes^6^. In our previous study, individual GFP-tagged TβRII molecules were imaged on the cell membrane using total internal reflection fluorescence microscopy (TIRFM) to study receptor activation. We demonstrated that TβRII occurs as a monomer in the resting state, and dimerizes upon TGF-β1 stimulation^7^, which supports the idea that the receptor dimerization is essential for receptor activation.

Here, we identified a direct interaction between metformin and TGF-β1 using a surface plasmon resonance (SPR)-based assay, and found that metformin inhibits [^125^I]-TGF-β1 binding to its receptor. We modeled the interaction mode between metformin and TGF-β1 using molecular docking and molecular dynamics simulations. We further investigated the binding force between TGF-β1 and TβRII using atomic force microscopy (AFM) and the dimerization of TβRII upon TGF-β1 stimulation using single molecule fluorescence imaging. This study revealed a novel mechanism underlying the inhibition of the TGF-β1 signaling pathway by metformin that explains the beneficial effects of metformin against organ fibrosis and cancer progression.

## Results

### Metformin directly interacts with TGF-β1, inhibits binding to its receptor, and attenuates downstream signaling

**Figure 1:**
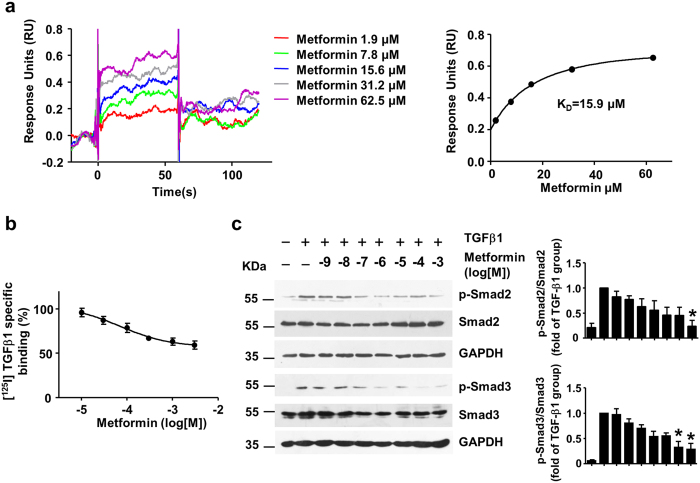
Metformin interacts with TGF-β1 to block its binding with receptors and attenuates downstream signaling (**a**) Sensorgrams for the binding of metformin and TGF-β1. TGF-β1 was covalently coupled to a CM5 chip, and metformin was injected in a two-fold dilution concentration series ranging from 62.5 μM to 1.9 μM. The steady-state values were calculated from the sensorgrams and plotted against the concentration. The data were fit to a single site binding model to calculate the K_D_. (**b**) Metformin inhibited [^125^I]-TGF-β1 receptor binding to 3T3 mouse fibroblasts. The results are expressed as the percentage of specific binding in the absence of metformin (n = 4). (**c**) Metformin and TGF-β1 (0.5 ng/mL) were premixed for 2 h and then 3T3 cells were treated with the mixture for 30 minutes. Western blot analysis and quantification of phosphorylated-Smad2 (p-Smad2), Smad2, p-Smad3, Smad3 and GAPDH were performed. Data are mean ± SEM from 4 independent experiments. Kruskal–Wallis ANOVA combined with post-hoc Dunn’s multiple comparison test (two tailed) was performed. *P < 0.05 vs. TGF-β1 group.

The SPR-based assay suggested that the binding of metformin to TGF-β1 occurred with a K_D_ value of 15.9 μM (Fig. 1a). Next, we measured the effect of metformin on the specific binding of [^125^I]-TGF-β1 to 3T3 fibroblasts. As shown in Fig. 1b, metformin inhibited [^125^I]-TGF-β1 binding to its receptor in 3T3 fibroblasts in a dose-dependent manner (log[IC50] = −4.16 ± 0.53). In contrast, HCT 116 cells that harbor a loss of function mutation in TβRII displayed negligible binding of [^125^I]-TGF-β1. And metformin has little effect on the [^125^I]-TGF-β1 binding to HCT 116 cells, suggesting that metformin inhibits the binding of [^125^I]-TGF-β1 to TβRII (see Supplementary Fig. S1). Consistent with these results, western blotting analyses indicated that metformin decreased TGF-β1-induced phosphorylation of Smad2 and Smad3 (pSmad2 and pSmad3) in 3T3 fibroblasts (Fig. 1c, Kruskal–Wallis ANOVA combined with post-hoc Dunn’s multiple comparison test, TGF-β1+ Metformin 0.1 mM vs. TGF-β1, pSmad3, P^*^ = 0.016; TGF-β1+ Metformin 1 mM vs. TGF-β1, pSmad2, P* = 0.041, pSmad3, P* = 0.017) and H9C2 cells (see Supplementary Fig. S2). Notably, metformin didn’t inhibit TGF-β1 induced Smad2/3 phosphorylation until 30 minutes after treatment (see Supplementary Fig. S3).

### Metformin attenuates TGF-β1 downstream signaling through AMPK independent pathway

**Figure 2:**
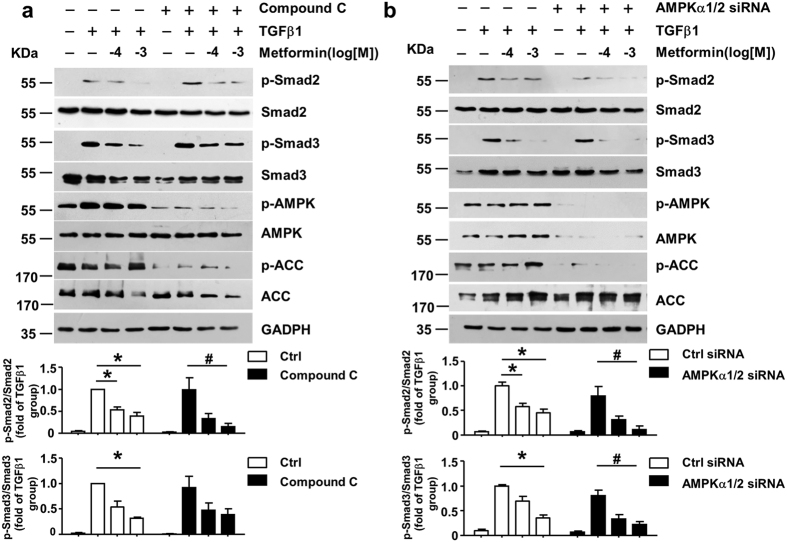
Metformin attenuates TGF-β1 downstream signaling through AMPK independent pathway (**a**) AMPK inhibitor, Compound C (5 μM) were pretreated in 3T3 cells for 4 h. n = 5. (**b**) Negative control siRNA and AMPKα1/2 siRNA (80 nM) were transfected in 3T3 cells for 48 h. n = 6. Next, metformin and TGF-β1 (0.5 ng/mL) were premixed for 2 h and then the 3T3 cells were treated with the mixture for 30 minutes. Western blot analysis and quantification of phosphorylated-Smad2 (p-Smad2), Smad2, p-Smad3, Smad3, p-AMPK, AMPK, p-ACC, ACC and GAPDH were performed. Data are mean ± SEM. Kruskal–Wallis ANOVA combined with post-hoc Dunn’s multiple comparison test (two tailed) was performed. *P < 0.05 vs. TGF-β1 group, ^#^P < 0.05 vs. TGF-β1 plus compound C or TGF-β1 plus AMPKα1/2 siRNA group.

Metformin is a well-known AMP-activated protein kinase (AMPK) activator. Phosphorylation of AMPK and Acetyl-CoA carboxylase (ACC), an AMPK target, were both decreased in AMPK inhibitor Compound C pretreatment group (Fig. 2a) and AMPKα1/2 siRNA transfected group (Fig. 2b), demonstrating the loss of AMPK activity. Notably, metformin still decreased p-Smad2/3 induced by TGF-β1, to a degree similar with that in the vehicle treatment group (Fig. 2a,b). This result indicated that metformin attenuates TGF-β1-induced p-Smad2/3 independent of AMPK pathway.

### Metformin antagonizes TGF-β1 signaling via a direct interaction with TGF-β1

**Figure 3:**
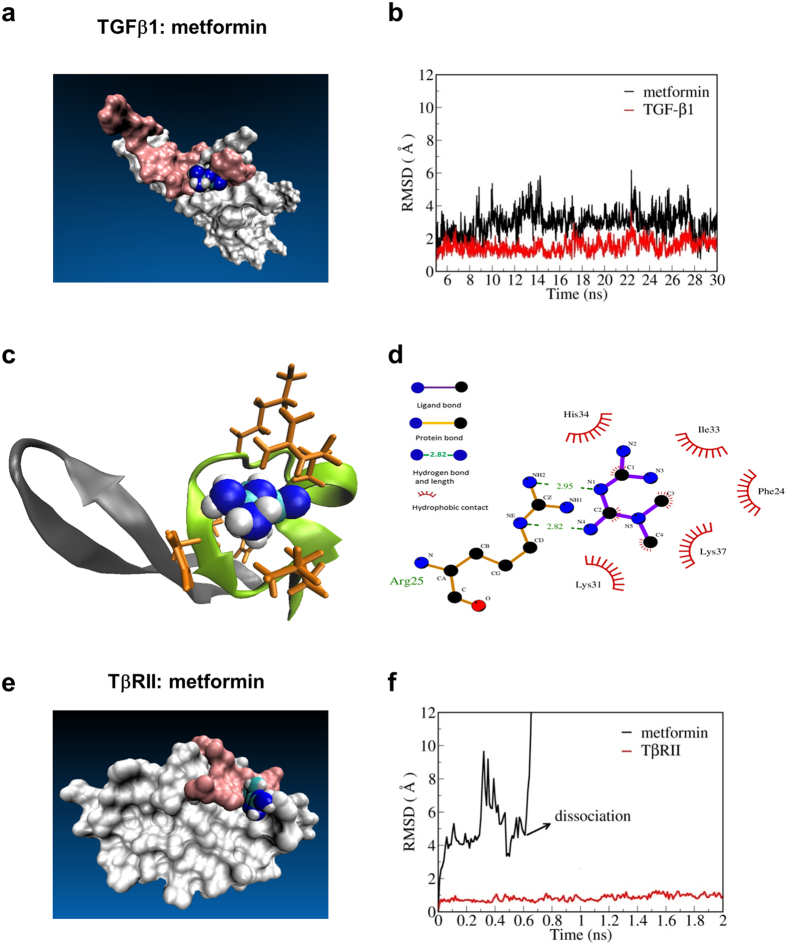
Molecular docking and molecular dynamics simulations for TβRII :metformin and TGF-β1:metformin (**a**) Structure of the TGF-β1:metformin complex after relaxation. The surface region of TGF-β1 recognized by type II TGF-β receptors (TβRII) is shown in pink. (**b**) Root-mean-square deviation (RMSD) of TGF-β1 and metformin relative to TGF-β1 during the last 25-ns trajectory. (**c**) Binding site of metformin consists of the β-sheet1 and β-sheet2 of TGF-β1. (**d**) Residues in direct contact with metformin (depicted using the LIGPLOT program with a cutoff of 3.9 Å). (**e**) Structure of the TβRII: metformin complex after relaxation. The surface region of TβRII recognized by TGF-β1 is shown in pink. (**f**) RMSD of TβRII and metformin relative to TβRII.

To further assess the potential binding of metformin to TGF-β1 and its receptor, we performed molecular docking and molecular dynamics (MD) simulation. The binding of metformin to TGF-β1 was stable, determined by the root mean square deviation (RMSD) of metformin relative to TGF-β1 (Fig. 3a,b). Metformin tended to bind in a cave-like structure consisting of the β-strand1 and β-strand2 of TGF-β1 (Fig. 3c). Importantly, this site was partially overlaid with the binding interface of TβRII. The residues in direct contact with TGF-β1 are depicted in Fig. 3d. The binding of metformin was largely attributed to the shape complementarity and hydrogen bond interaction between the guanidine group and Arg25. In addition, the nonpolar components (methyl groups) of metformin were nestled in the hydrophobic bottom of the cave. Thus, the binding stability of metformin to TGF-β1 was further evaluated according to the binding free energy using Molecular Mechanics/Poisson Boltzmann Surface Area methods. The estimated value of the binding free energy (*ΔG*_*bind*_) was −68.50 kJ/mol, which was considered to be sufficiently strong for such a small compound. However, metformin could not stably bind to the putative binding site of TβRII(extracellular domain) (Fig. 3e). The molecule quickly diffused away from the initial binding site during the molecular simulation (Fig. 3f).

### Metformin inhibits the binding probability of TGF-β1 and TβRII but has no effect on the binding force of TGF-β1 and TβRII

**Figure 4:**
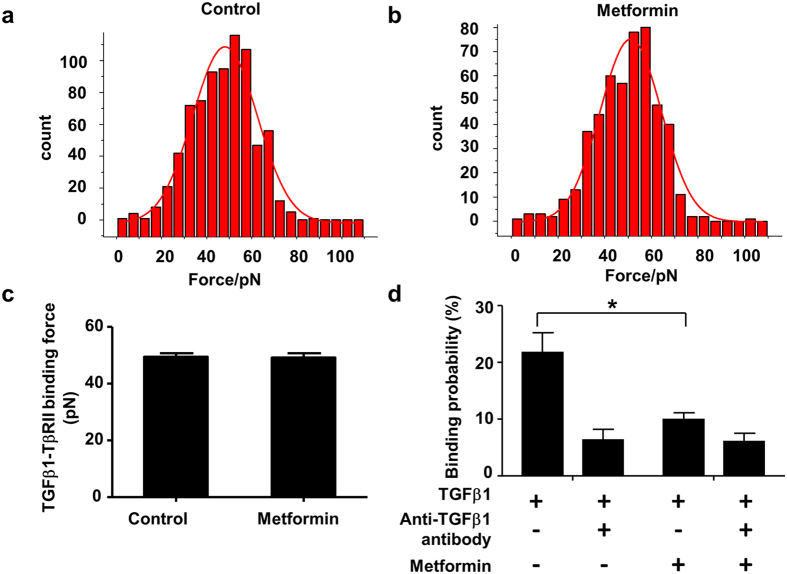
Metformin inhibits the binding probability but not binding force of TGF-β1 and TβRII. TGF-β1–TβRII binding force measurements were obtained with TGF-β1-modified atomic force microscopy (AFM) tips on HeLa cells, which express TβRII. Blocking experiments were performed by the addition of free TGF-β1 monoclonal antibodies into the solution. (**a**) Histograms of binding forces of TGF-β1/TβRII in the untreated cells and (**b**) the cells treated with 50 μM metformin. (**c**) Binding forces of TGF-β1/TβRII in cells treated with/without 50 μM metformin. (**d**) Binding probability of TGF-β1and TβRII when the cells were treated with metformin and/or the blocking reagent (anti-TGF-β1 antibody). Data were expressed as the mean ± SEM from 3 independent experiments. Mann-Whitney U test (one tailed) with exact method was used. *P < 0.05.

To determine how metformin inhibits the binding of TGF-β1 to its receptor, we next performed TGF-β1–TβRII binding force measurements on live cells using AFM-based single-molecule force spectroscopy. Blocking experiments were performed by the addition of free TGF-β1 monoclonal antibodies. As shown in Fig. 4a,b, the force distribution histogram displayed a single maximum by a Gaussian fit and the binding probability was less than 30%, indicating that the single molecule forces were measured. In the cells treated with metformin (50 μM), similar binding forces (measured as the averaged histogram peak value) were observed for TGF-β1 with TβRII on the cell surface as the control (Mann-Whitney U test with exact method, control vs. metformin: 49.5 ± 1.3 vs.49.3 ± 1.4 pN, P > 0.05, Fig. 4c). However, metformin decreased the binding probabilities from 21.7 ± 3.5% to 9.9 ± 1.2%, which was similar to the result of the TGF-β1 antibody treatment (6.4 ± 1.9%, Fig. 4d).

### Metformin inhibits TGF-β1-induced TβRII dimerization

**Figure 5:**
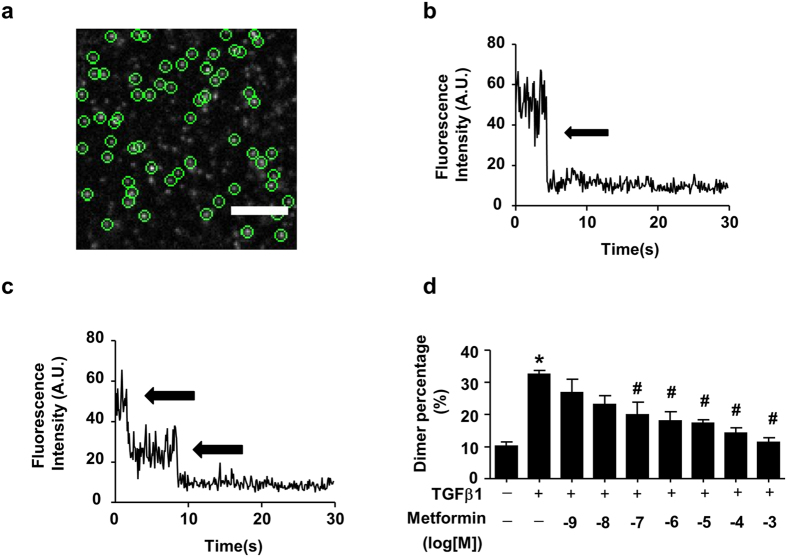
Metformin inhibits TGF-β1-induced TβRII dimerization. (**a**) Typical single-molecule image of TβRII-GFP on the HeLa cell membrane. After transfection with TβRII-GFP for 4 h, HeLa cells were imaged using total internal reflection fluorescence microscopy (TIRFM). The diffraction-limited spots (5 × 5 pixel regions) enclosed with green circles represent the signals from individual TβRII-GFP molecules, and they were chosen for the intensity analysis. Scale bar, 5 μm. (**b**,**c**) Two representative time course graphs of GFP emissions after background correction demonstrating one- and two-step bleaching, respectively. The arrows indicate the bleaching steps. The individual TβRII-GFP molecules were monomers when they were bleached in one step (**b**) and dimers when they were bleached in two steps (**c**). (**d**) Metformin inhibited TGF-β1-induced TβRII dimerization as shown by single-molecule imaging. Fraction of two-step bleaching events for TβRII-GFP molecules (counted spots were set as 100%) was represented as the dimer percentage. Prior to single-molecule fluorescence imaging, metformin and TGF-β1 (10 ng/mL) were premixed for 2 h, and HeLa cells were then treated with the mixture for 15 min at 37 °C. The data were presented as the mean ± SEM (n = 5–16). ANOVA combined with Tukey’s post-hoc test (two tailed) was used. *P < 0.05 vs. control group, ^#^P < 0.05 vs. TGF-β1 group.

Because TGF-β1-induced TβRII dimerization is a consequence of TGF-β ligand–receptor interaction and essential for receptor activation^7^, we next determined the effect of metformin on the formation of ligand-induced TβRII dimers. By analyzing the photobleaching traces (Fig. 5a), we found that 88.8% (778 out of 876 spots from 14 fixed cells) of individual TβRII-GFP molecules were monomers because they bleached in one step (Fig. 5b), 10.7% (94 of 876 spots) were dimers because they bleached in two steps (Fig. 5c), and that 0.5% (4 of 876) bleached in three steps. Following the TGF-β1 stimulation, 67.7% (529 of 781 spots) bleached in one step as monomers, 31.6% (247 of 781spots from sixteen fixed cells) bleached in two steps as dimers and 0.6% (5 of 781) bleached in three steps. As shown in Fig. 5d, metformin inhibited the percentage of dimers induced by TGF-β1 in a dose-dependent manner.

## Discussion

In the present study, we demonstrated that metformin antagonized TGF-β1 signaling by interacting with the TGF-β1 ligand, thereby blocking the binding of TGF-β1 to TβRII and resulting in decreased downstream signaling. This finding provides new indications for metformin, including numerous TGF-β1 hyperfunction-associated diseases. Based on the interaction between metformin and TGF-β1, new compounds with similar properties could be further developed.

It is generally accepted that metformin acts via the activation of AMPK and the inhibition of mitochondrial respiratory-chain complex 1^8^. Our previous study has shown that metformin inhibits TGF-β1 induced collagen synthesis independent of AMPK activation^5^. Here, we further discovered that metformin antagonizes TGF-β1 signaling by directly binding to TGF-β1 which is independent of AMPK activation. Consistent with the well-established role of TGF-β1 in the exacerbation of fibrosis^9^, our previous study and other studies have shown that metformin treatment attenuates cardiac fibrosis^5^, liver fibrosis^10^ and renal fibrosis^11^. In addition, metformin has been shown to inhibit TGF-β1-induced EMT which plays a key role in carcinoma progression and organ fibrosis^12^,^13^. Moreover, clinical trials have suggested that metformin is associated with decreased cancer risk and improved prognosis in cancer patients^14^,^15^. These findings support the idea that metformin exerts a protective effect against organ fibrosis and malignant tumor progression by blocking TGF-β1.

In addition to fibrosis and tumors, TGF-β is involved in numerous other diseases. Thus, targeting the TGF-β signaling pathway has become attractive for drug development. Currently, therapeutic strategies against the TGF-β family include three approaches: 1) inhibition at the translational level using antisense oligonucleotides, 2) inhibition of the ligand-receptor interaction using ligand traps and anti-receptor monoclonal antibodies, and 3) inhibition of the receptor-mediated signaling cascade using inhibitors and aptamers of TGF-β receptor kinases^9^,^16^. However, these approaches have specific challenges that limit their application, such as the limited ability of an antisense oligonucleotides and monoclonal antibodies to reach the targeted tissue^17^,^18^. In contrast, metformin is a small molecule compound that can easily reach the targeted tissue. Inhibitors of TGF-β receptor kinases have side effects that occur through the potential cross-inhibition of other kinases^9^. Conversely, metformin has been shown to be safe and have fewer side effects over decades of use. In addition, metformin has beneficial effects beyond targeting TGF-β1 and based on the interaction mode between metformin and TGF-β1, additional compounds can be developed to target TGF-β with higher specificity and potency.

In summary, our study identified metformin as a novel TGF-β1 suppressor, and this action underlies the pleiotropic effects of the drug. This finding strongly supports the clinical use of metformin as a treatment for numerous diseases beyond diabetes where TGF-β1 signaling malfunctions are indicated. In addition, our study provides insights that can be used in the development of new compounds targeting TGF-β1.

## Methods

### Reagents

Metformin was purchased from Sigma-Aldrich (St. Louis, MO, USA). Compound C was purchased from EMD Millipore Corp. (Billerica, MA, USA). Recombinant human TGF-β1 was purchased from PeproTech (Rocky Hill, NJ, USA). [^125^I]-TGF-β1 was purchased from Perkin Elmer Inc. (Waltham, MA, USA).

### Equipment and settings

For single molecule fluorescence imaging, a custom-built TIRFM system was used as previously described^7^. The experiment was performed on TIRFM with 100X/1.45NA Plan Apochromat TIR objective (Olympus, Japan) and a 14-bit back-illuminated electron-multiplying charge-coupled device camera (Andor iXon DU-897 BV). Imaging was performed at room temperature. GFP was excited at 488-nm by an argon laser (Melles Griot,Carlsbad, CA, USA) with the power of 1 mW measured after the laser passing through the objective. Movies of 200–300 frames were acquired for each sample at a frame rate of 10 Hz. Sequences of images were stored directly to a computer hard drive for subsequent analysis by IQ live cell imaging software (Andor Technology, BT, UK).

### SPR spectroscopy

Experiments were performed at 25 °C using a Biacore T200 and the data were analyzed using Biacore T200 evaluation software 2.0 (GE Healthcare, Stockholm, Sweden). TGF-β1 was covalently coupled to a CM5 chip (GE Healthcare) and metformin was injected in a two-fold dilution concentration series ranging from 62.5 μM to 1.9 μM. The steady-state values were calculated from the sensorgrams and plotted against the concentrations. The data were fit to a single site binding model to calculate the K_D_.

### TGF-β1 binding assay

The binding experiments were performed as previously described^19^. Briefly, 3T3 cells (American Type Culture Collection, ATCC^®^ CRL-2752™) or HCT 116 cells (American Type Culture Collection, ATCC^®^ CRL-247™) were seeded onto a 24-well-plate and cultured in DMEM supplemented with 10% Fetal Bovine Serum and antibiotics (100 U/mL penicillin-streptomycin). When cells were at a near-confluent stage, 50 pM [^125^I]-TGF-β1 with or without different concentrations of metformin were added. After 4 h at 4 °C, the medium was removed and cells were washed five times with ice-cold binding buffer (50 mM HEPES, 128 mM NaCl, 5 mM KCl, 5 mM MgSO4, and 1.2 mM CaCl2). The cells were then solubilized using binding buffer containing 1%Triton X-100 and the radioactivity was measured. Non-specific binding was determined in the presence of unlabeled TGF-β1 (10 nM).

### Western blotting analysis

Western blotting analyses were performed using specific antibodies as previously described^5^. Total proteins were extracted from 3T3 cells or H9C2 cells (American Type Culture Collection, ATCC^®^ CRL-1446™) by use of RIPA buffer (6.5 mM Tris, pH 7.4, 15 mM NaCl, 1 mM EDTA, 0.1% SDS, 0.25% sodium deoxycholate, 1% NP-40). Bicinchoninic acid reagents were used to measure the protein concentration. Equal amounts of proteins were separated by SDS-PAGE and transferred to nitrocellulose filter membranes. Phospho-Smad2 Ser465/467 (3108S), Smad2 (5339S), phospho-Smad3 Ser423/425 (9520S), Smad3 (9513S), phospho-AMPKα Thr172 (2531S), AMPKα (2532S), phosphor-ACC Ser79 (3661S), and ACC (3662S) antibodies were purchased from Cell Signaling Technology (Danvers, MA, USA). GAPDH antibodies were purchased from Santa Cruz Biotechnology (sc-47724, Santa Cruz, CA, USA). The blots were immunoreacted with primary antibodies (4 °C, 16 h) and secondary antibodies conjugated with horseradish peroxidase (room temperature, 1 h). Protein bands were visualized by enhanced chemiluminescence detection and the intensity was quantified by use of Image-J software.

### siRNA knockdown

3T3 fibroblasts at 30% confluence were transfected with AMPKα1/2 siRNA (sc-45313, Santa Cruz Biotechnology, Santa Cruz, CA, USA), or negative control siRNA (1027281, QIAGEN, Valencia, CA, USA) at 80 nM with HiPerFect transfection reagent (301705, QIAGEN, Valencia, CA, USA). Experiments were performed with these cells at 48 h post-transfection.

### Theoretical study: molecular docking and molecular dynamics simulation

The geometry structure of metformin was optimized with Hartree-Forck methods at 6–31 + G* level of theory. The crystal structures of TGF-β1 and the extracellular domain of TβRII, were retrieved from the PDB archives (3KFD)^20^. Autodock4.2 suite^21^ was first applied to predict the preferential binding poses of ligand (metformin) in both TGF-β1 and TβRII. Then the structure of both TGF-β1 and TβRII bound with metformin were obtained for further evaluation by MD simulation. Amber99SB-ILDN forcefield^22^ for protein and General Amber force field^23^ for ligand was used. The charge parameters of ligand were taken from restrained electrostatic potential calculation^24^. The protein-ligand complex was solvated with TIP3P water. Sodium and Chloride ions were added to neutralize the system. All simulations were carried out with the GROMACS4.6.1 packages^25^ and were run in NPT ensemble. The temperature (T = 300k) and pressure (p = 1atm) was kept constant using velocity scaling methods and Berendsen barostat methods, respectively. Based on the results of simulation, Molecular Mechanics/Poisson Boltzmann Surface Area methods^26^ was used to estimate the binding free energy of metformin on protein.

### Single molecule fluorescence imaging

Hela cells (American Type Culture Collection, ATCC^®^ CCL-2™) were transfected with TβRII-GFP plasmid for 4 h. Prior to the single-molecule fluorescence imaging, the cells were treated with TGF-β1 (10 ng/ml for 15 min) and different concentrations of metformin and then washed twice and fixed. The single-molecule fluorescence intensity and photobleaching steps were also analyzed as previously reported^7^.

### AFM tips preparation and AFM force measurements

TGF-β1-modified AFM tips (type: NP-10, Bruker, Santa Barbara, CA, USA) were prepared as previously reported^27^. Hela cells were transfected with the TβRII-GFP plasmid for 24 h, and the force measurements were performed on a PicoSPM II system with a PicoScan 3000 controller and a large scanner (Agilent, Santa Clara, CA, USA). The AFM scanner was mounted on an inverted fluorescence microscope (Olympus IX71, Japan). The fluorescent protein-labeled cells were used to guide the AFM tips on the cell expressing TβRII. All of the force curves were measured with the contact mode at room temperature using a soft cantilever (0.06 N m^−1^). The loading rate of the force measurements was 1.0 × 10^4^ pN/s. The force curves were recorded using PicoScan 5 software (Molecular Imaging, Tempe, AZ) and analyzed using a user-defined program in MATLAB (MathWorks Corp., Beijing, China).

### Statistical Analysis

Data were expressed as the means ± SEM from at least 3 independent experiments. In parametric data, Student’s *t* test or ANOVA combined with Tukey’s post-hoc test was used to analyze the differences among groups if data were determined to be normal distribution by K-S test. For non-parametric data, Mann-Whitney U test with exact method was used to analyze the differences between two groups. A Kruskal–Wallis ANOVA combined with post-hoc Dunn’s multiple comparison test was performed when more than 2 groups were evaluated. A *p-*value < 0.05 was considered statistically significant.

## Additional Information

**How to cite this article**: Xiao, H. *et al*. Metformin is a novel suppressor for transforming growth factor (TGF)-β1. *Sci. Rep.* **6**, 28597; doi: 10.1038/srep28597 (2016).

## Supplementary Material

Supplementary Information

## References

[b1] El Messaoudi S., Rongen G. A. & Riksen N. P. Metformin therapy in diabetes: the role of cardioprotection. Curr Atheroscler Rep. 15, 314 (2013). 23423523 10.1007/s11883-013-0314-z

[b2] Tang T. & Balen A. H. Use of metformin for women with polycystic ovary syndrome. Hum Reprod Update. 19, 1 (2013). 23114640 10.1093/humupd/dms040

[b3] Nasri H. . Bright renoprotective properties of metformin: beyond blood glucose regulatory effects. Iran J Kidney Dis. 7, 423–428 (2013). 24241085

[b4] Leone A., Di Gennaro E., Bruzzese F., Avallone A. & Budillon A. New perspective for an old antidiabetic drug: metformin as anticancer agent. Cancer Treat Res. 159, 355–376 (2014). 24114491 10.1007/978-3-642-38007-5_21

[b5] Xiao H. . Metformin attenuates cardiac fibrosis by inhibiting the TGFbeta1-Smad3 signalling pathway. Cardiovasc Res. 87, 504–513 (2010). 20200042 10.1093/cvr/cvq066

[b6] Moustakas A. & Heldin C. H. The regulation of TGFbeta signal transduction. Development. 136, 3699–3714 (2009). 19855013 10.1242/dev.030338

[b7] Zhang W. . Single-molecule imaging reveals transforming growth factor-beta-induced type II receptor dimerization. Proc Natl Acad Sci USA 106, 15679–15683 (2009). 19720988 10.1073/pnas.0908279106 PMC2747179

[b8] Foretz M., Guigas B., Bertrand L., Pollak M. & Viollet B. Metformin: from mechanisms of action to therapies. Cell Metab. 20, 953–966 (2014). 25456737 10.1016/j.cmet.2014.09.018

[b9] Akhurst R. J. & Hata A. Targeting the TGFbeta signalling pathway in disease. Nat Rev Drug Discov. 11, 790–811 (2012). 23000686 10.1038/nrd3810 PMC3520610

[b10] Duvnjak M. . Therapy of nonalcoholic fatty liver disease: current status. J Physiol Pharmacol. 60 Suppl 7, 57–66 (2009). 20388946

[b11] Cavaglieri R. C., Day R. T., Feliers D. & Abboud H. E. Metformin prevents renal interstitial fibrosis in mice with unilateral ureteral obstruction. Mol Cell Endocrinol. 412, 116–122 (2015). 26067231 10.1016/j.mce.2015.06.006

[b12] Cufi S. . Metformin against TGFbeta-induced epithelial-to-mesenchymal transition (EMT): from cancer stem cells to aging-associated fibrosis. Cell Cycle. 9, 4461–4468 (2010). 21088486 10.4161/cc.9.22.14048

[b13] Thiery J. P., Acloque H., Huang R. Y. & Nieto M. A. Epithelial-mesenchymal transitions in development and disease. Cell. 139, 871–890 (2009). 19945376 10.1016/j.cell.2009.11.007

[b14] Soranna D. . Cancer risk associated with use of metformin and sulfonylurea in type 2 diabetes: a meta-analysis. Oncologist. 17, 813–822 (2012). 22643536 10.1634/theoncologist.2011-0462 PMC3380880

[b15] Rizos C. V. & Elisaf M. S. Metformin and cancer. Eur J Pharmacol. 705, 96–108 (2013). 23499688 10.1016/j.ejphar.2013.02.038

[b16] Sheen Y. Y., Kim M. J., Park S. A., Park S. Y. & Nam J. S. Targeting the transforming growth factor-beta signaling in cancer therapy. Biomol Ther (Seoul). 21, 323–331 (2013). 24244818 10.4062/biomolther.2013.072 PMC3825194

[b17] Nagaraj N. S. & Datta P. K. Targeting the transforming growth factor-beta signaling pathway in human cancer. Expert Opin Investig Drugs. 19, 77–91 (2010). 10.1517/13543780903382609 PMC2796203 20001556

[b18] Chames P., Van Regenmortel M., Weiss E. & Baty D. Therapeutic antibodies: successes, limitations and hopes for the future. Br J Pharmacol. 157, 220–233 (2009). 19459844 10.1111/j.1476-5381.2009.00190.x PMC2697811

[b19] Murakami S. . Ursolic acid, an antagonist for transforming growth factor (TGF)-beta1. FEBS Lett. 566, 55–59 (2004). 15147868 10.1016/j.febslet.2004.04.036

[b20] Radaev S. . Ternary complex of transforming growth factor-beta1 reveals isoform-specific ligand recognition and receptor recruitment in the superfamily. J Biol Chem. 285, 14806–14814 (2010). 20207738 10.1074/jbc.M109.079921 PMC2863181

[b21] Morris G. M. . AutoDock4 and AutoDockTools4: Automated docking with selective receptor flexibility. J Comput Chem. 30, 2785–2791 (2009). 19399780 10.1002/jcc.21256 PMC2760638

[b22] Lindorff-Larsen K. . Improved side-chain torsion potentials for the Amber ff99SB protein force field. Proteins. 78, 1950–1958 (2010). 20408171 10.1002/prot.22711 PMC2970904

[b23] Wang J., Wolf R. M., Caldwell J. W., Kollman P. A. & Case D. A. Development and testing of a general amber force field. J Comput Chem. 25, 1157–1174 (2004). 15116359 10.1002/jcc.20035

[b24] Bayly C. I., Cieplak P., Cornell W. D. & Kollman P. A. A well-behaved electrostatic potential based method using charge restraints for deriving atomic charges - the resp model. J. Phys Chem. 97, 10269–10280 (1993).

[b25] Hess B., Kutzner C., van der Spoel D. & Lindahl E. GROMACS 4: Algorithms for highly efficient, load-balanced, and scalable molecular simulation. J Chem Theory Comput. 4, 435–447 (2008). 26620784 10.1021/ct700301q

[b26] Srinivasan J., Cheatham T. E., Cieplak P., Kollman P. A. & Case D. A. Continuum solvent studies of the stability of DNA, RNA, and phosphoramidate - DNA helices. J Am Chem Soc. 120, 9401–9409 (1998).

[b27] Yu J. . Single-molecule force spectroscopy study of interaction between transforming growth factor beta1 and its receptor in living cells. J Phys Chem B. 111, 13619–13625 (2007). 17997544 10.1021/jp0758667

