# Supplementary material for: Metformin is a novel suppressor for transforming growth factor (TGF)-β1
Source: Sci Rep. 2016 Jun 28;6:28597. doi: 10.1038/srep28597 (PMC4923858; doi:10.1038/srep28597)

Supplementary Information

Metformin is a novel suppressor for transforming growth factor (TGF)-β1

Han Xiao, Jianshu Zhang, Zhonghe Xu, Yenan Feng, Mingliang Zhang, Jianli Liu,

Ruifei Chen, Jing Shen, Jimin Wu, Zhizhen Lu, Xiaohong Fang, Jingyuan Li,

Youyi Zhang*

Supplementary Figure legends

Figure S1. Effects of metformin on [^{125}I]-TGF-β1 binding to 3T3 fibroblasts and TβRII mutated HCT 116 cells 50 pM [^{125}I]-TGF-β1 with or without different concentrations of metformin were added in the cells. After 4 h at 4 °C, cells were washed and solubilized in the buffer. The radioactivity was then measured. Data are mean ± SEM from 4 independent experiments.

Figure S2. Metformin attenuates TGF-β1 downstream signaling in H9C2 cells Metformin and TGF-β1 (0.5 ng/mL) were premixed for 2 h and then H9C2 cells were treated with the mixture for 30 minutes. (a)Western blot analysis and quantification of phosphorylated-Smad2 (p-Smad2), Smad2, and GAPDH were performed. (b) Western blot analysis and quantification of p-Smad3, Smad3 and GAPDH were performed. Data are mean ± SEM from 4 independent experiments. Kruskal–Wallis ANOVA combined with post-hoc Dunn's multiple comparison test (two tailed)

was performed. *P < 0.05 vs. TGF-β1 group.

Figure S3. Metformin didn't attenuate TGF-β1 induced Smad2/3 phosphorylation until 30 min after treatment Metformin (1 mmol/L) and TGF-β1 (0.5 ng/mL) were premixed for 2 h and then 3T3 fibroblasts were treated with the mixture for 1, 5, 15, and 30 minutes. (a)Western blot analysis and quantification of phosphorylated-Smad2 (p-Smad2), Smad2, and GAPDH were performed. (*P < 0.05; n = 4). (b) Western blot analysis and quantification of p-Smad3, Smad3, and GAPDH were performed. (*P < 0.05; n = 5). Data are mean ± SEM. Student's *t* test (two tailed) was used to analyze the differences between TGF-β1 and TGF-β1 plus metformin groups.

Supplementary Figure S1

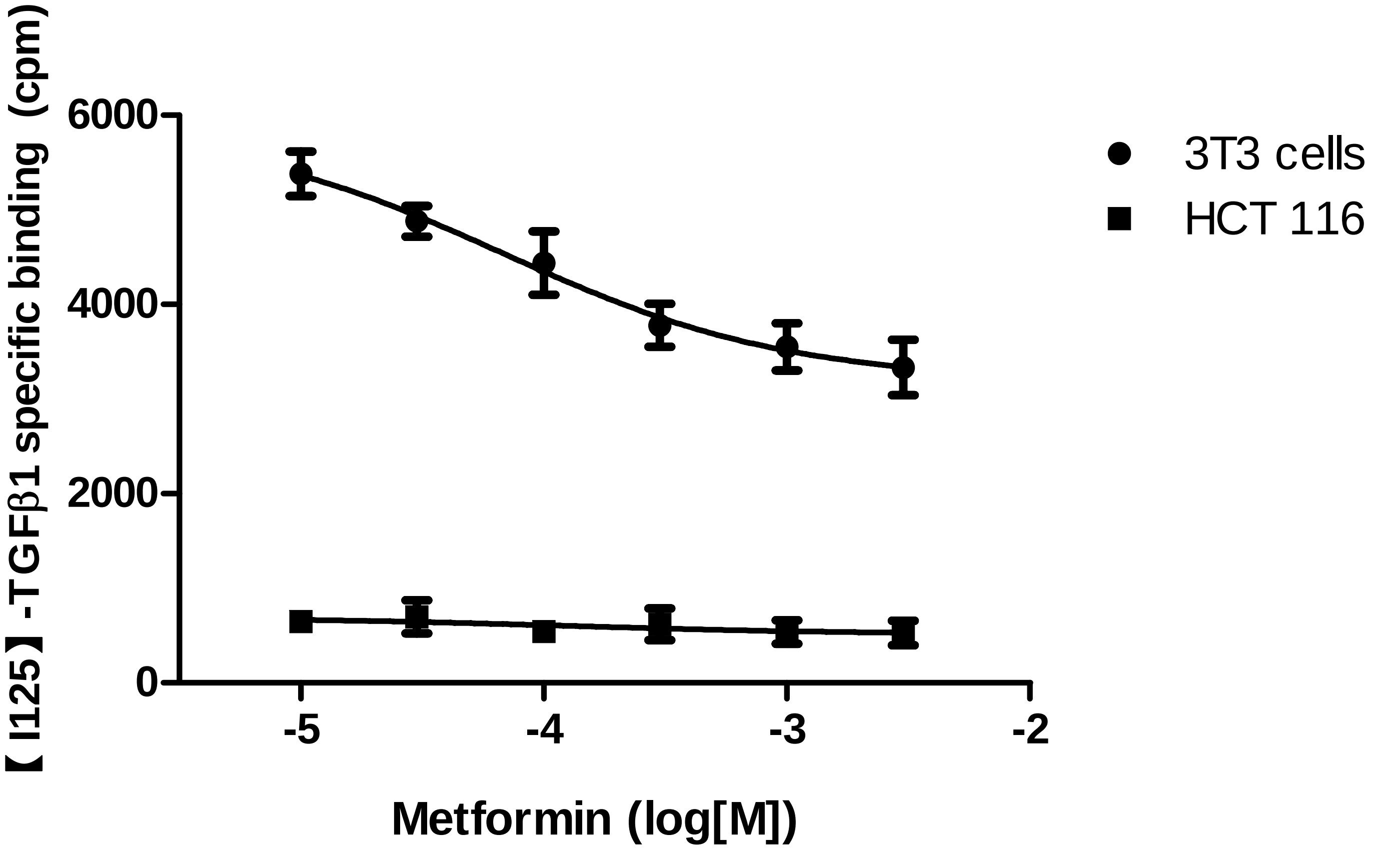

Supplementary Figure S2

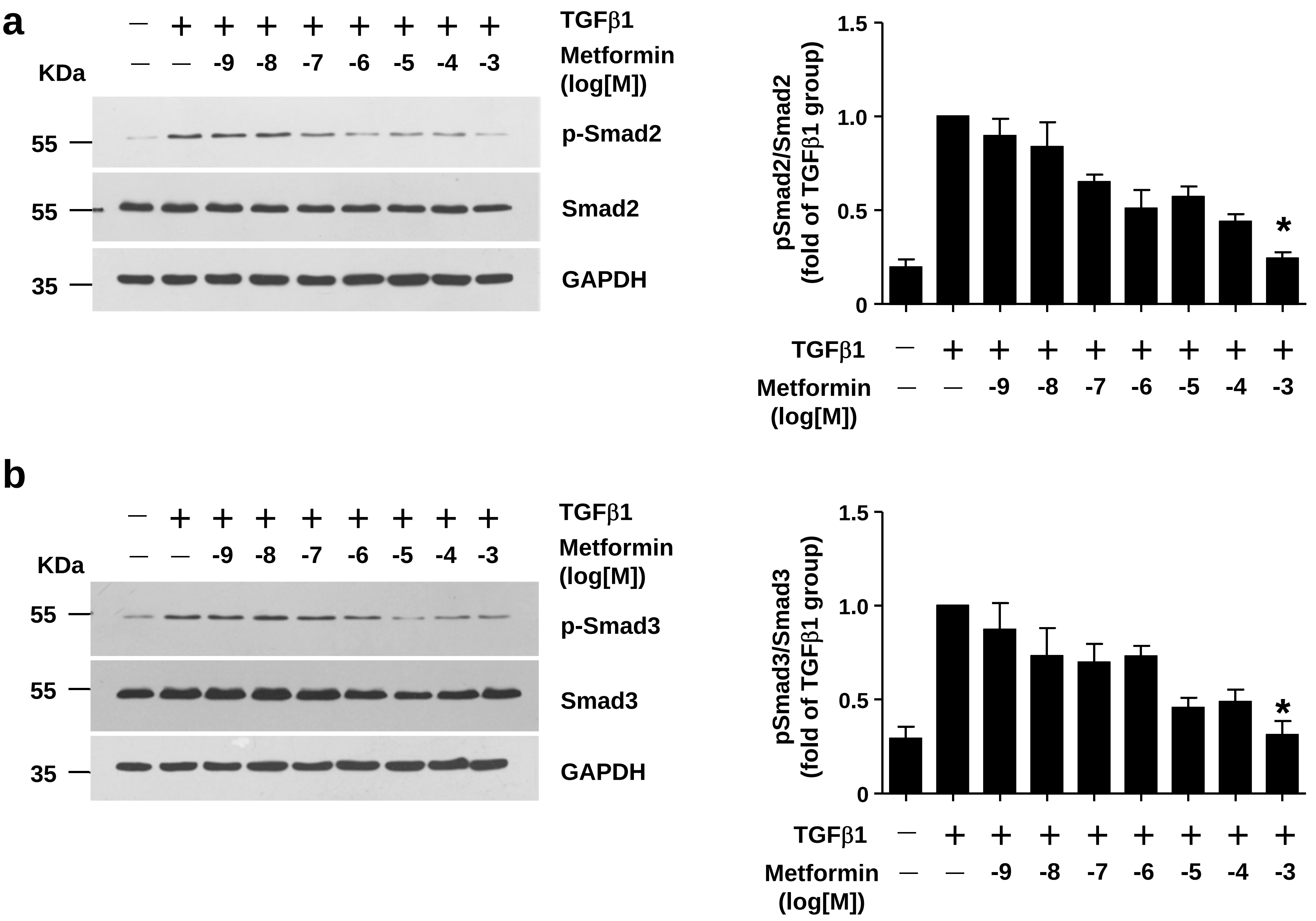

Supplementary Figure S3

a

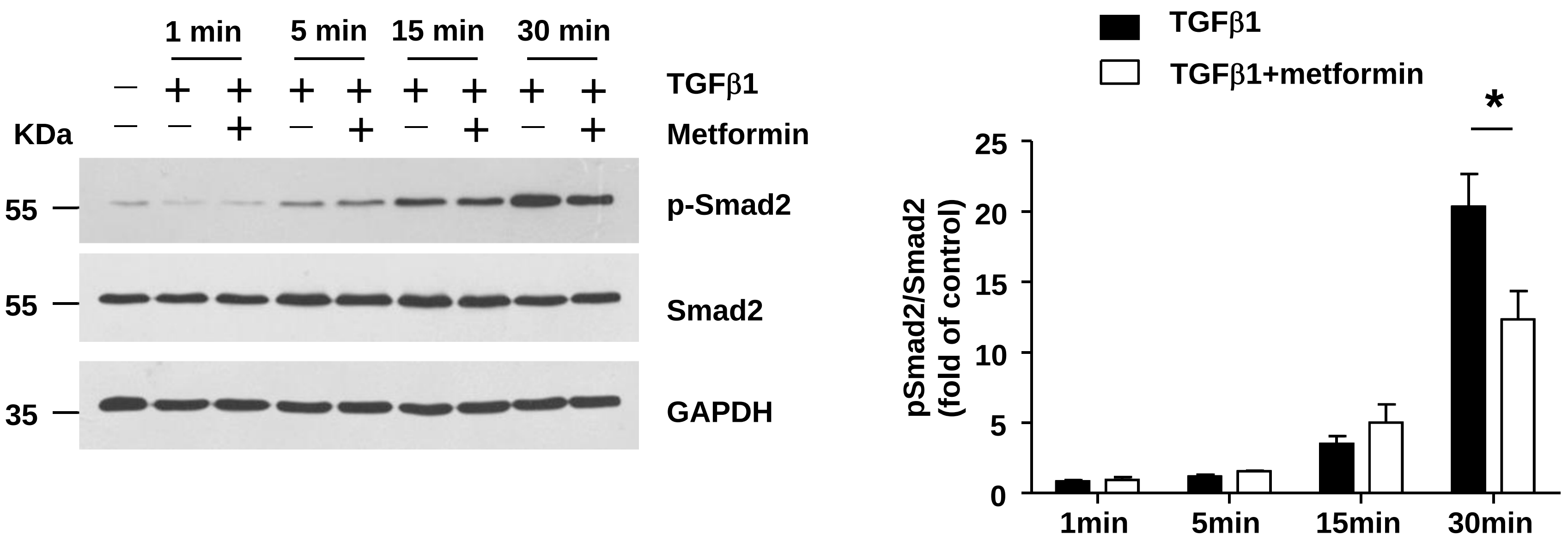

b

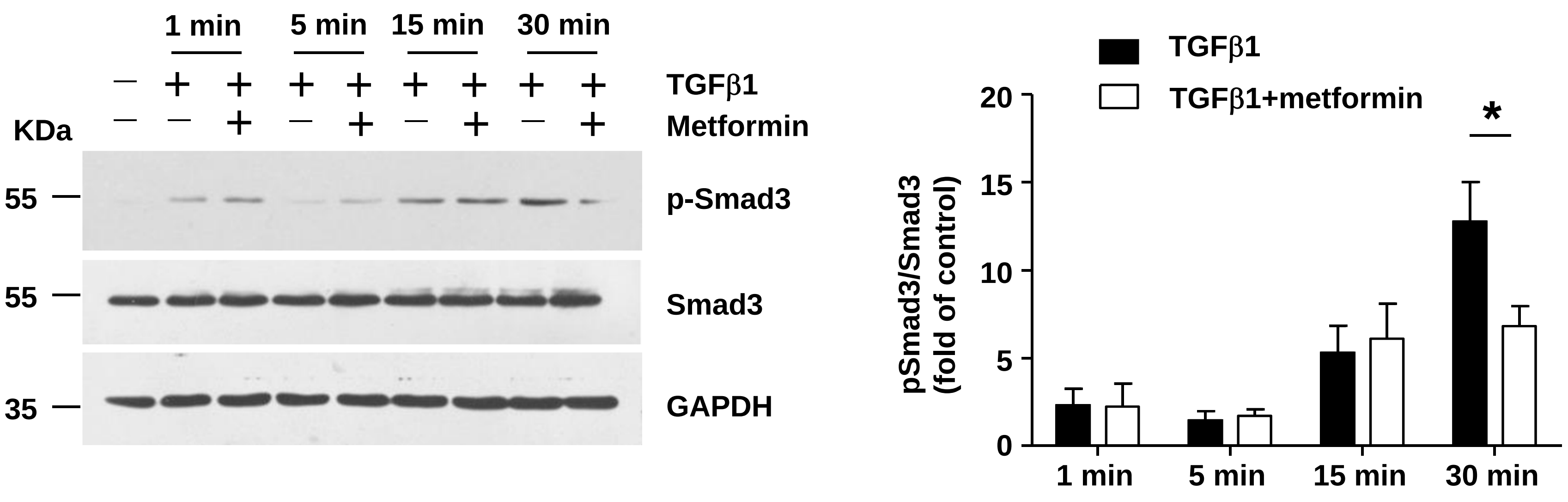

Supplement: Supplementary Information [file srep28597-s1.pdf]
